# Soluble PD-L1 in NSCLC Patients Treated with Checkpoint Inhibitors and Its Correlation with Metabolic Parameters

**DOI:** 10.3390/cancers12061373

**Published:** 2020-05-27

**Authors:** Angelo Castello, Sabrina Rossi, Luca Toschi, Luigi Mansi, Egesta Lopci

**Affiliations:** 1 Nuclear Medicine, Humanitas Clinical and Research Hospital—IRCCS, 20089 Rozzano (MI), Italy; angelo.castello@humanitas.it; 2 Medical Oncology, Humanitas Clinical and Research Hospital—IRCCS, 20089 Rozzano (MI), Italy; sabrina.rossi@humanitas.it (S.R.); luca.toschi@humanitas.it (L.T.); 3 Section Health and Development, Interuniversity Research Center for Sustainability (CIRPS), 80100 Naples, Italy; mansi.luigi@libero.it

**Keywords:** non-small cell lung cancer, circulating PD-L1, positron emission tomography, immunotherapy, response, outcome

## Abstract

We investigated the role of soluble PD-L1 (sPD-L1) in non-small cell lung carcinoma (NSCLC) patients treated with immune checkpoint inhibitors (ICI) and analyzed its association with clinical outcomes and metabolic parameters by 18F-fluorodeoxyglucose positron emission tomography/computed tomography (18F-FDG-PET/CT). Between July 2017 and May 2019, we enrolled 20 candidate patients of ICI therapy who had serum frozen samples and 18F-FDG PET/CT available, both at baseline and at the first response evaluation. This analysis is embedded into a larger prospective study (NCT03563482). Twelve out of 20 patients received nivolumab, one patient received combination of nivolumab and ipilimumab, whereas the others received pembrolizumab. Median sPD-L1 level at baseline was 27.22 pg/mL. We found a significant association between patients with elevated sPD-L1, above the median value, and high metabolic tumor burden, expressed by metabolic tumor volume (MTV, 115.3 vs. 35.5, *p* = 0.034) and total lesion glycolysis (TLG, 687 vs. 210.1, *p* = 0.049). At the first restaging after 7–8 weeks, median sPD-L1 levels significantly increased as compared to baseline median value (43.9 pg/mL, *p* = 0.017). No significant differences in response rates were detected, according to both morphological and metabolic response criteria. Likewise, no difference in survival outcomes were observed between low sPD-L1 and high sPD-L1 patients. The increase of sPD-L1 concentrations during ICI treatment may reflect the expansion of tumor volume and the tumor lysis. Moreover, it is supposed that sPD-L1 has its own biological action, either by reducing membrane PD-1 sites available for nivolumab or by inducing lymphocytes exhaustion after binding their membrane PD-1. Further, larger studies are needed to confirm our preliminary results on the role of sPD-L1 during ICI therapy.

## 1. Introduction

Lung cancer is the first cause of cancer-related death worldwide, with a poor prognosis especially in the advanced stages [1]. Recently, the advent of immune checkpoint inhibitors (ICI) has broaden therapeutic options available for oncologists. In particular, by blocking programmed cell death (PD-1)/programmed death ligand (PD-L1) interaction or other checkpoints, e.g., cytotoxic T-lymphocyte-associated protein 4 (CTLA-4), ICI restore immune surveillance against malignant cells and improve clinical responses and survival in many tumor types [2,3]. Despite the success, only a small portion of patients benefit from these new therapies. Thus, the research for reliable predictive factors discriminating responders from non-responders is a priority, although it is still scarce and under debate [4,5].

Beside tumor-tissue biomarkers, such as membrane PD-L1 expression and mutational burden, the identification of potential blood-based biomarkers has attracted the attention of medical community. Indeed, blood draw has the advantage of being easy to perform, allowing continuous evaluation during therapy. Nevertheless, most studies have identified unspecific parameters so far, e.g., LDH, neutrophils or lymphocytes, whereas few data exist regarding specific ICI-related proteins [6]. With this regard, soluble PD-L1 (sPD-L1) levels have been explored in several tumors with contrasting results [7,8,9]. Moreover, there are still some aspects on which to shed light about the role and function of sPD-L1, particularly in connection to ICI setting. For example, the source of sPD-L1 remains uncertain, whether from malignant or immune cells or both. Likewise, the two forms, i.e., bound and soluble, might show different binding capacities for their ligand.

Based on the above evidence, we investigated the role of sPD-L1 in NSCLC patients treated with ICI and their association with metabolic parameters by 18F-FDG PET/CT.

## 2. Results

### 2.1. Baseline sPD-L1 and Patients’ Characteristics

**Figure 1:**
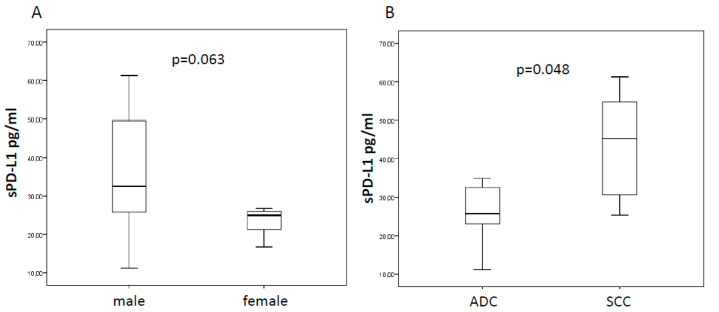
Box plots for sPD-L1 according to gender (female vs. male) and tumor histotypes (squamous cell carcinoma compared to adenocarcinoma; ADC vs. SCC). In the first case (**A**), the median values of sPD-L1 showed a trend towards statistical significance (24.98 vs 32.55 pg/mL), while the significant difference was confirmed for histology (45.28 vs. 25.68 pg/mL; (**B**)).

**Figure 2:**
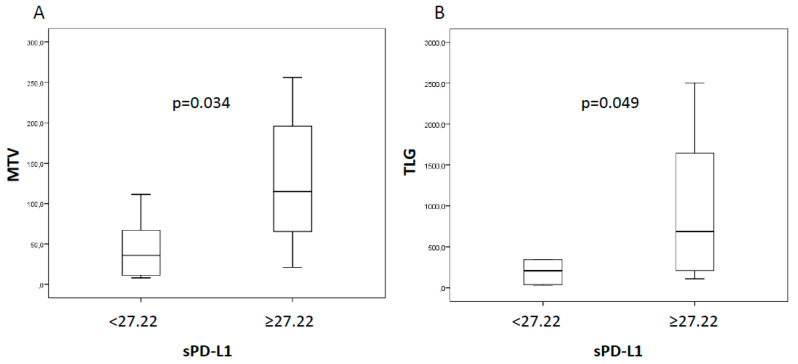
Box plots for MTV (**A**) and TLG (**B**) in patients with low or high sPD-L1 levels at baseline documenting a significant difference in median values: MTV, 115.3 vs. 35.5 and TLG, 687 vs. 210.1.

Among 20 patients who met the inclusion criteria, 12 received nivolumab, seven pembrolizumab, while only one patient received combination of nivolumab and ipilimumab. The majority (60%) exhibited an Eastern Cooperative Oncology Group (ECOG) performance status score of 0. Almost all patients were smokers (85%) and the adenocarcinoma was the histological tumor type most represented (70%). ICI therapy was started as first line treatment in six out of 20 (30%) patients, whereas 14 (70%) patients received one or more lines of treatment before ICI. Median sPD-L1 level at baseline was 27.22 pg/mL (range 11.23–61.27). We evaluated the potential association between baseline sPD-L1 levels and clinic-pathological characteristics (Table 1). Notably, we observed a trend toward a decrease of sPD-L1 in women, even if it was not statistically significant (24.98 vs. 32.55 pg/mL, *p* = 0.063, Figure 1A). Moreover, we found significantly higher sPD-L1 concentration into the bloodstream of squamous cell carcinoma than adenocarcinoma (45.28 vs. 25.68 pg/mL, *p* = 0.048, Figure 1B). Additionally, we assessed the association between sPD-L1 and metabolic parameters extracted by 18F-FDG PET/CT. Of note, we found that patients with elevated sPD-L1, above the median value, had a greater metabolic tumor burden, expressed by metabolic tumor volume (MTV, 115.3 vs. 35.5, *p* = 0.034) and total lesion glycolysis (TLG, 687 vs. 210.1, *p* = 0.049) (Figure 2).

Finally, no correlation was found between sPD-L1 levels and patients’ factors, such as age, neutrophil to lymphocyte ratio (NLR), previous treatment, and tumor PD-L1 expression.

### 2.2. sPD-L1, Tumor Response, and Survival

At the first restaging, after 3–4 cycles of ICI (Table 1), median sPD-L1 levels significantly increased as compared to baseline median value (43.9 pg/mL, *p* = 0.017).

According to iRECIST (Immune Response Evaluation Criteria in Solid Tumors), two patients (10%) experienced partial response (PR), 11 (55%) had a stable disease (SD), and 7 (35%) progression disease (PD), while imPERCIST (Immunotherapy-modified PET response criteria in solid tumors) identified one patient (5%) with a complete metabolic response (CMR), four (20%) with partial metabolic response (PMR), 11 (55%) with stable metabolic disease (SMD), and four (20%) with progression metabolic disease (PMD). However, no significant difference in sPD-L1 levels have been observed among response rates.

At a median follow-up 10.3 months (range 2–29), progression disease occurred in 15 (75%) patients, 12 (60%) patients died because of tumor progression, whereas eight (40%) patients were still alive at the time of analysis. Median progression-free survival (PFS) was 5.6 months and median overall survival (OS) was 13.2 months. No difference in PFS nor in OS were observed in patients with low sPD-L1 levels compared to those with elevated sPD-L1 at baseline (PFS: 4 vs. 5.8 months, *p* = 0.595; OS: 8.1 vs. 18.8 months, *p* = 0.483). Likewise, no differences in PFS and OS were detected at the first restaging after 7–8 weeks.

**Figure 3:**
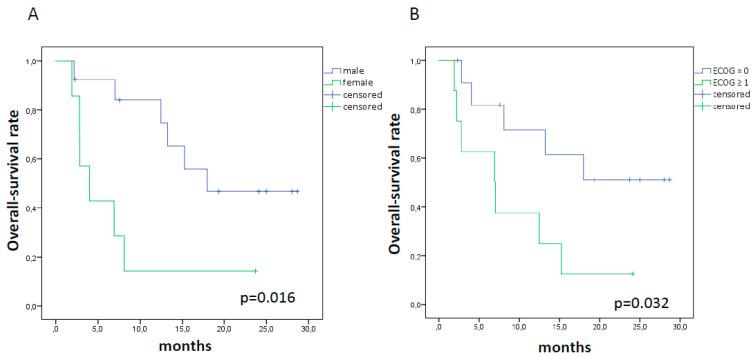
Kaplan Meier curves for OS in female patients ((**A**); 4 vs. 18 months, *p* = 0.016) and in those with ECOG score at baseline ≥ 1 ((**B**); 6.9 vs. median not reached, *p* = 0.032).

Among clinical-pathological variables, we observed a shorter OS in female patients (4 vs. 18 months, *p* = 0.016) and in those with ECOG score at baseline ≥ 1 (6.9 vs. median not reached, *p* = 0.032), while PFS rates were not significantly different (Figure 3).

## 3. Discussion

The detection of reliable predictive biomarkers has become a “hot topic” for treatment with ICI, at least since the recognition of antithetical responses in patients with apparently similar clinical characteristics. Most of ICI approved so far are based on PD-L1 cell surface expression, although its effective predictive value remains under debate due to tumor heterogeneity and lack of standardized methods. Therefore, several studies have been focusing on new potential biomarkers, considering cancer, immunological, and host characteristics as unique entities, ranging from tumor immuno-phenotypes to circulating molecules and immunological profiles [10,11,12].

In the present study, we have added another piece in the big puzzle of immunotherapy. Indeed, by using the ELISA test, we demonstrated an interesting association between sPD-L1 levels and elevated metabolic tumor burden, suggesting a larger release of malignant particles into the bloodstream from extended tumors. This is in line with a recent study of Vecchiarrelli and colleagues, who demonstrated a positive correlation between sPD-L1 levels and metastatic sites [13]. Even though this finding need to be confirmed in a larger cohort, the combination of sPD-L1 levels along with metabolic parameters could be useful for treatment optimization, allowing to identify non-responder patients already at baseline, as demonstrated by our group and others [14,15]. Previously, we have documented the potential prognostic role of combined sPD-L1 and metabolic parameters already in NSCLC candidate patients of surgery [16]. In the present study, we found that females have a significantly lower level of sPD-L1 before ICI therapy. Previously, Conforti et al. [17] have assessed the relationship between gender and efficacy of ICI. Their meta-analysis suggested that men have a significantly reduced risk of death than women. In our cohort females had shorter OS than males, thus our study opens an interesting interrogative on the reason why women have lower sPD-L1 levels. Some hypothesis can be argued, for example sex-hormone regulation of the expression and function of PD-1/PD-L1 or other mechanisms of immune escape adopted by tumors in women [18].

Interestingly, in our cohort, a significant difference in sPD-L1 was observed in patients based on tumor histotypes. In particular, median values resulted higher for squamous cell carcinoma compared to adenocarcinoma, 45.28 vs. 25.68 pg/mL, respectively (*p* = 0.048; Figure 1B). Previously, Okuma et al [19] had investigated the sPD-L1 in the blood stream of advanced lung cancer patients reporting no correlation for the sPD-L1 plasma levels with histological subtypes. Conversely, Cheng and colleagues [20] have proven that lung adenocarcinoma patients have higher levels of sPD-L1 than patients with squamous cell carcinoma. These conflicting results may be partially explained by the differences in treatment lines between our cohort, mostly pre-treated prior to ICI, and patients investigated by the Chinese group [20]. In fact, although we did not find any significant influence from previous chemotherapy in sPD-L1 levels, we know from other studies [13] that an increase in sPD-L1 from baseline can be seen in patients receiving chemotherapy, but not in patients undergoing other treatment types [21].

In the current study, we showed in addition a significant variation of median sPD-L1 after three or four cycles of ICI, which was consistent with previous studies [22,23]. The origin and function of sPD-L1 are still scarcely known, some potential biological elucidations on the efficacy of immunotherapy can be discussed. On one hand, the increase of sPD-L1 concentrations during ICI treatment may reflect the expansion of tumor volume and the tumor lysis. On the other hand, it is supposed that sPD-L1 has its own biological action, either by competing and reducing membrane PD-1 sites available for nivolumab or by inducing lymphocytes exhaustion and tumor evasion after binding their membrane PD-1 [21,24].

The absence of a statistically significant association between sPD-L1 levels and response rates or survival may be a result of the small patients analyzed, compared to other studies [23,24]. Another reason may be the relatively high number of patients (approximately 55%) included within SD/SMD group by both response criteria adopted. Indeed, SD/SMD patients are a quite heterogeneous group including both non-responders, displaying a lack of sensitivity to treatment, and responders, who might not reach the cut-off value to be classified as PR/PMR.

Finally, some limitations need to be mentioned. Apart from the small size of the study, we did not measure plasma levels of PD-1, which were analyzed in previous studies [23,25]. Moreover, tumor PD-L1 expression was available for only 11 patients, and therefore we were not able to assess the relationship between soluble and tumor PD-L1.

## 4. Materials and Methods

### 4.1. Patients and Study Design

Between July 2017 and May 2019, we prospectively enrolled 20 patients (13 males, 7 females, mean age 72 years) with NSCLC candidate to ICI therapy at our Institution Humanitas Clinical and Research Center. We included in our analysis patients who had serum frozen samples and 18F-FDG PET/CT available, both at baseline and at the first restaging after approximately three or four cycles of ICI. Nivolumab was administered intravenously every two weeks at dosage of 3 mg/kg, while pembrolizumab every three weeks at 200 mg. One patient received ipilumumab 1 mg/Kg in combination with nivolumab, This analysis is embedded into a larger prospective study (NCT03563482). The study was conducted in accordance with the Declaration of Helsinki Declaration and approved by a local medical ethical board (Prot. Nr. CE Humanitas ex D.M. 8/2/2013 335/17). All patients provided written informed consent before the enrollment.

### 4.2. Clinical Endpoints 

The main objective of the study was to explore the relationship between sPD-L1 and clinical characteristics, including metabolic parameters by 18F-FDG PET/CT, and how the expression of sPD-L1 levels might be modified by ICI treatment.

Secondary objective of the study was to investigate the relationship between sPD-L1 levels of NSCLC patients and clinical outcomes, including progression-free survival (PFS) and overall survival (OS). Tumor response was evaluated using iRECIST and imPERCIST criteria [26,27]. PFS and OS were defined as time from therapy start until disease progression or death, respectively.

### 4.3. Quantification of Circulating PD-L1 

Before and after 3–4 cycles of ICI, peripheral blood samples were collected from patients included in the study according to standardized protocol (Humanitas Centre for Biological Resource Standard Operating Procedures), centrifuged for 15 min and frozen at −80 ℃. The expression levels of PD-L1 were assessed in plasma using commercial ELISA kit (PD-L1/CD274, Quantikine ELISA, R&D Systems, Inc, Minneapolis, MN, USA) and following the manufacturer’s instruction. The results were obtained using a spectrophotometer (Microplate Absorbance Reader, Biorad, Italy) reading at 450 nm, and concentrations of PD-L1 were calculated according to standard curves.

### 4.4. Imaging Protocol and Tumor Delineation

In fasting patients for at least 6 h, 18F-FDG were administered with an activity between 250 and 500 MBq. After approximately 60 min from radiopharmaceutical administration, images were acquired using two dedicated PET/CT scanners (Siemens Biograph LSO and GE Discovery PET/CT 690), accredited by EANM Research Ltd (EARL) program. Images were reconstructed on a GE ADW4.6 workstation (GE Healthcare, Waukesha, WI, USA) and interpreted by experienced nuclear medicine physicians. 18F-FDG PET/CT images were interpreted visually and semi-quantitatively. SUVmax was obtained by generating a 3D volume of interest on the basis of the tumor-related activity volume by applying a percentage threshold = 41%. MTV and TLG were computed for each metabolic lesion. TLG was calculated as the product of MTV and SUVmean.

### 4.5. Statistical Analysis

Serum PD-L1 levels were not normally distributed as tested by Kolmogorov–Smirnov normality test, and thus they were indicated as median and range. The Mann–Whitney U test or the Wilcoxon matched pairs test was used for the comparison of two specifications. The Kruskal–Wallis test was used for the comparison of three or more specifications. A relative change in sPD-L1 concentration was determined by dividing the sPD-L1 concentration after 3–4 cycles of ICI treatment by that at diagnosis. PFS and OS were analyzed using the Kaplan–Meier method. The log-rank test was used for comparison of survival between groups. Median follow-up time was calculated as time between onset of ICI treatment and death or last patient contact. All statistical analyses were carried out using the Statistical Package for Social Sciences, version 23.0, for Windows (SPSS, Chicago, IL, USA), and *p* values < 0.05 were considered to be statistically significant.

## 5. Conclusions

To summarize, we demonstrated the association between metabolic tumor burden and sPD-L1 levels, as well as a significant increase of sPD-L1 during treatment with ICI. Our data have allowed us to take another small step in the complex world of immune responses, using sPD-L1 as a new biomarker in the early assessment and monitoring of immunotherapy efficacy. Nevertheless, in future, larger studies and a longer follow-up are needed to deepen our understanding of the role of sPD-L1.

## Figures and Tables

**Table 1:** Clinical characteristics, soluble programmed death-ligand 1 (sPD-L1) levels, and outcomes for all study patients.

Patient	Gender (M/F)	Age	ICI		sPD-L1		PFS Months	OS Months
				Pre-	Post-	%		
1	F	76	pembrolizumab	24.98	61.45	146.00	4.0	8.1
2	M	86	nivolumab	58.54	46.30	−20.91	6.5	15.2
3	M	79	nivolumab	25.86	39.69	53.48	12.1	13.2
4	F	77	pembrolizumab	56.52	49.56	−12.31	1.5	2.8
5	F	55	nivolumab	23.04	31.41	36.33	1.7	2.8
6	M	65	pembrolizumab	27.71	47.53	71.53	28.0	28.0
7	M	79	nivolumab	49.64	41.89	−15.61	5.8	7.0
8	M	70	nivolumab	24.18	19.60	−18.94	5.0	12.4
9	M	80	nivolumab	54.75	77.84	42.17	4.9	7.5
10	F	78	nivolumab	16.69	45.41	172.08	23.7	23.7
11	M	70	nivolumab + ipilimumab	30.61	54.40	77.72	23.2	24.1
12	M	52	nivolumab	34.93	51.49	47.41	1.6	2.2
13	F	77	nivolumab	25.33	28.85	13.90	6.0	6.9
14	M	73	pembrolizumab	61.27	27.18	−55.64	28.7	28.7
15	M	51	nivolumab	40.92	50.44	23.26	2.3	2.3
16	M	78	nivolumab	32.55	42.33	30.05	5.6	18.0
17	M	53	pembrolizumab	11.23	26.56	136.51	1.8	19.4
18	F	80	nivolumab	26.74	21.89	−18.14	1.8	4.0
19	F	80	pembrolizumab	19.51	64.71	231.68	0.1	1.9
20	M	75	pembrolizumab	25.50	42.33	66.00	25.0	25.0
